# Agreement between pre-post measures of change and transition ratings as well as then-tests

**DOI:** 10.1186/1471-2288-13-52

**Published:** 2013-03-27

**Authors:** Thorsten Meyer, Susanne Richter, Heiner Raspe

**Affiliations:** 1 Integrative Rehabilitation Research Unit, Institute for Epidemiology, Social Medicine and Health Systems Research, OE5410, Hannover Medical School, Carl-Neuberg-Str. 1, Hannover, 30165, Germany; 2 Institute for Social Medicine, University of Luebeck, Luebeck, Germany; 3 Population Medicine, University of Luebeck, Luebeck, Germany

## Abstract

**Background:**

Different approaches have been developed for measuring change. Direct measurement of change (transition ratings) requires asking a patient directly about his judgment about the change he has experienced (reported change). With indirect measures of change, the patients’ status is assessed at different time points and differences between them are calculated (measured change). When using the quasi-indirect approach (‘then-test’), patients are asked after an intervention to rate their statuses both before the intervention as well as at the time of the enquiry. Associations previous studies have found between the different approaches might be biased because transition ratings are generally assessed using a single, general item, while indirect measures of change are generally based on multi-item scales. We aimed to quantify the agreement between indirect and direct as well as indirect and quasi-indirect measures of change while using multi-item scales exclusively. We explored possible reasons for non-agreement (present-state bias, recall bias).

**Methods:**

We re-analysed a data set originally collected to investigate the prognostic validity of different approaches of change measurements. Patients from a 3-week inpatient rehabilitation programme for either cardiac or musculoskeletal disorders filled in health-status questionnaires (which included scales for sleep function, physical function, and somatisation) both at admission and at discharge. The patients were then randomised to receive either an additional transition-rating or then-test questionnaire at discharge.

**Results:**

Out of 426 patients, 395 (92.7%) completed all questionnaires. Correlation coefficients between indirect and quasi-indirect measures of change ranged from *r* = .60 to *r* = .71, compared to *r* = .37 to *r* = .48 between indirect and direct measures of change. Correlation coefficients between pre-test and retrospective pre-test (then-test) results ranged from *r* = .69 to *r* = .82, indicating a low level of recall bias. Pre-test variation accounted for a substantial amount of variance in transition ratings in addition to the post-test scores, indicating a low level of present-state bias.

**Conclusions:**

Indirect and quasi-indirect measurements of change yielded comparable results indicating that recall bias does not necessarily affect quasi-indirect measurement of change. Quasi-indirect measurement might serve as a substitute for pre-post measurement under conditions still to be specified. Transition ratings reflect different aspects of change than indirect and quasi-indirect methods do, but are not necessarily biased by patients’ present states.

## Background

A valid measurement of change is a prerequisite for evaluating health outcomes. From a clinical perspective, observing change over the course of a patient’s disease is a crucial part of the treatment process. From a research perspective, it is important to know whether, to what extent and how any observed changes are causally related to medical interventions. On the level of the healthcare system, the effectiveness of healthcare measures has to be demonstrated or continually monitored, e.g. for quality assurance programmes.

**Figure 1:**
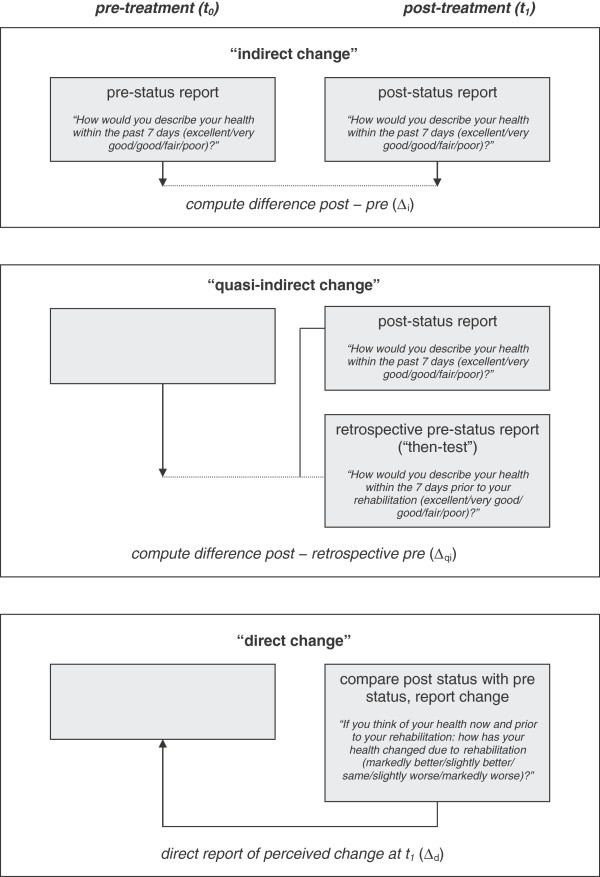
**Approaches to the measurement of change, incl. examples.** Legend: t_0_ = pre treatment (admission), t_1_ = post treatment (discharge).

Different approaches for measuring change have been developed. A) Clinicians often rely on ‘direct’ measures of change, also referred to as ‘transition ratings’. To assess change directly, clinicians either form an impression of how much the patient’s complaints or symptoms have changed, or they solicit the patient’s judgment of this change directly (e.g. “Has your leg pain improved, stayed the same, or worsened?”). B) In clinical studies, however, ‘indirect’ measures of change are preferred. To determine change indirectly, researchers assess a patient’s status at different points in time and obtain measures of observed change by calculating the respective differences (deltas) between measurement points. C) Another important approach for measuring change has emerged in quality-of-life research. It has been shown that patients’ response shifts may bias results of clinical studies if the internal criteria or metric they base their responses on change in the time interval between the two responses used to calculate change [1-3]. The ‘then-test’ method has been developed to take this response-shift phenomenon into account. Patients are asked at t1 not only to rate their current status at t1 but also retrospectively to rate their status at t0 (hence the ‘then-test’ designation). The assumption is that response shift is eliminated because the patient will have used the same metric for both the t0 and t1 ratings since they were assessed at the same time. The researcher then calculates the delta between the t1 rating and the retrospective t0 rating. We will refer to this type of change measurement as ‘quasi-indirect’ [4]. Figure 1 depicts these three common approaches to measuring change and provides examples for each of them.

From a naïve perspective, one might assume that all three approaches to measuring change should yield similar results because they should all measure the same change process. However, a number of studies have only been able to detect low to moderate correlations between indirect and direct (transition ratings) measures of change. Although studies exist reporting correlation coefficients in the *r* = 0 to *r* = 0.40 range [5], there are also studies reporting correlation coefficients well above *r* = 0.60 [6] and even above *r* = 0.80 [7]. There are very few studies evaluating the correlation between indirect and quasi-indirect measures of change, despite a considerable number of studies on the response-shift phenomenon and the then-test approach [3]. In one sample of frail, elderly patients accessing community-based rehabilitation services, the correlation coefficients between indirect and quasi-indirect measures of change were moderate to low: intra-class-correlations of *ICC* = 0.41 and *ICC* = 0.21 were reported for the EQ-5D utility score and general health perception (visual analogue scale), respectively [8].

To our knowledge, there are no studies available that would explain this lack of agreement^a^. However, there are a number of theoretical reasons why different biases might affect the different approaches to measure change.

Recall bias: With direct measures of change, patients have to recall a specified prior state and compare it with their present state in order to come up with a transition rating. With quasi-indirect measures of change, patients have to recall a specified prior state and give it an explicit rating. These memories of past states are known to be biased [9,10]. That being said, there are empirical studies that have found substantial associations between pre-status reports and “then-test” measures [6,7].

Present state effect: It has been postulated that patients use their present state to judge whether or how much they have changed, i.e. patients’ assessments of change would be unduly influenced by their present states. For example, if a person feels well at the time of measurement, he might infer that his status has improved, or vice versa, without actually having taken his prior state into account. In fact, transition ratings have been shown to be highly correlated to post-treatment ratings [11,12]. Guyatt et al. have argued that if an assessment of change using transition ratings is unbiased, then post scores and pre scores should correlate with direct measures of change, with equal magnitude and opposite direction [7]. Empirical studies have repeatedly shown transition ratings to be more strongly correlated to post-status scores than to pre-status scores [9,13]. That said, pre-status scores have usually been able to account for additional variance in transition ratings when used second to post-status scores [7].

There is a major drawback in the way that current studies are interpreted. Transition ratings are usually elicited on an aggregate level, i.e. single items are used to cover a whole domain of a construct of interest. For example, general transition ratings (also called ‘global perceived-effect scales’; *cf*. [8]) are used routinely to measure change directly (e.g. [9-11]), while multi-item scales are routinely used when measuring change indirectly. There are different theoretical reasons for why change measurements based on multi-item scales could differ from those based on general or aggregate transition ratings. We assume that there could be a substantial difference between the constructs the multi-item scales are intended to represent and those constructs that are evoked in a patient confronted with a single general term in a transition rating, i.e. these multi-item and general item assessments may refer to different aspects of the construct because of their differing levels of abstraction [5,14]. For example, a multi-item scale measuring functional disability and a single general question on functional disability might not evoke the same associations in the patient being questioned. From a psychometric perspective, multi-item scales should be more reliable than single-item measures [15].

The aim of the present study was to analyse the level of agreement between indirect and direct as well as indirect and quasi-indirect measures of change by using multi-item scales for all three approaches (including direct measures). Specifically, we aim to analyse 1) the level of agreement between direct and indirect as well as quasi-indirect and indirect measures of change, 2) how recall bias might account for differences between the performance of direct and quasi-indirect measures of change, and 3) how much the present-state effect affects direct measures of change.

## Methods

We re-analysed a data set originally collected to investigate the prognostic validity of different approaches for measuring change [16]. The original study had been motivated by the decision to use direct measurements of change in the quality-assurance programme for medical rehabilitation clinics under the purview of German statutory pension funds [17].

### Sample

Five rehabilitation clinics located in the German federal state of Schleswig-Holstein recruited study participants in 1999 (August to November) using the following inclusion criteria: (a) between 18–60 years old, (b) German speaking, (c) participating in a rehabilitation programme for either a musculoskeletal (ICD-9 710 to 739.9) or cardiovascular disease (ICD-9 393 to 429.9) at one of the five cooperating clinics.

**Figure 2:**
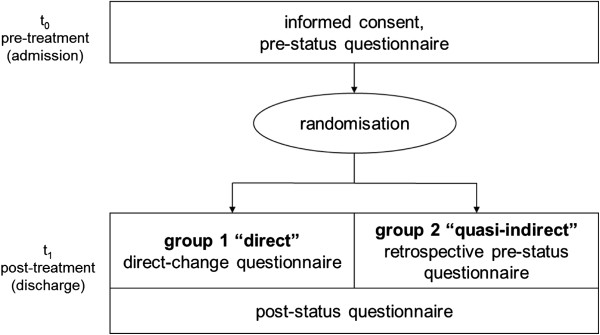
Study design.

Four hundred and twenty-six patients gave written, informed consent to participating in the study. They filled out a self-administered questionnaire both pre (before) treatment (t0; responding: *n* = 426, 100%) and post treatment (t1; responding: *n* = 397, 93.2%). At t1, all participants were randomised and asked to fill out one of two additional questionnaires, which were either designed to measure change directly (transition ratings) or quasi-indirectly (the “then-test” approach). In each clinic, participants were randomly allocated 1:1 either to group 1 (reporting change directly; responding: *n* = 194) or group 2 (reporting their pre status retrospectively; responding: *n* = 201). The standard duration of rehabilitation was three weeks, which represents the difference between t0 and t1. Figure 2 illustrates the study design.

The original study also included additional measurement points at follow-ups 6 and 12 months after t0 for the purpose of analysing predictive validity of the three different approaches of change measurements. These results are not part of the present analysis.

### Outcomes

Questionnaires at t0 and t1 gathered information on patients’ subjective health status (general health status, sleep, concentration, vitality, symptom checklist, pain, social functioning and physical functioning [18-21]). At t0, we assessed patients’ socio-demographic profile (age, sex, education, citizenship, marital status, net income), socio-medical characteristics (e.g. health insurance status, pension fund, healthcare utilisation, or any severe disabilities or disabilities currently preventing them from working), physical activities, risk factors alcohol/nicotine consumption, medications, height, and weight. We analysed 1) the four-item “sleep function” subscale of the IRES (Indicators of Rehabilitation Status; six response categories), which is a generic health-related quality-of-life measure widely used in German rehabilitation research and quality-assurance programs [18,19], 2) the ten-item “physical functioning index” subscale of the Short Form 36 (SF-36; three response categories) [20], and 3) the 12-item “somatisation” subscale of the Symptom Checklist 90-R (SCL-90-R; four response categories) [21]. These three scales were selected for clinical and psychometric reasons. Musculoskeletal and cardio-vascular diseases often involve somatisation, functional impairments, and insomnia [22,23]. The selected scales are reliable, valid and well-established for the assessment of subjective health of patients with musculoskeletal and cardiovascular diseases. These scales are included in the patient questionnaire used in the quality-assurance programme for medical rehabilitation clinics under the purview of German statutory pension funds [17].

Our re-analysis focused on these three scales because they were the only ones from the original study to apply all three methods of change measurement using the same number of items and featuring equivalent item content. An item of the sleep scale concerning disturbed sleep provides an illustrative example. Patients were asked about the extent to which their sleep was disturbed both before (t0) and after (t1) rehabilitation. At t1, they were also asked either how their problem they possibly had with their sleep being disturbed had changed (direct measurement of change) or to rate the extent to which their sleep had been disturbed at t0 (retrospective pre or then-test).

### Analysis

The differences in sample characteristics between the two randomized groups were analysed by means of *χ*^2^-tests and *t*-tests for independent samples, depending on their scale of measure.

In order to base all analyses of change on the same data, we included only those patients in the analyses who had provided valid data on the pre- and post-status scores in addition to providing either a retrospective pre score or a score for direct measurement of change for each of the three subscales (IRES sleep subscale, SF-36 physical functioning scale, SCL-90-R somatisation scale).

Three different change scores were calculated for each scale (“sleep function”, “physical functioning” and “somatisation”): The change scores for the indirect measures of change were calculated by subtracting the pre scale score at t0 from the post scale score at t1 (post − pre). The quasi-indirect measures of change were calculated by subtracting the retrospective pre-scale score referring to t0 from the post scale score at t1 (post − retrospective pre).

For each item, the response format for the direct measures of change comprised five categories (1 - markedly better, 2 - slightly better, 3 – no change, 4 - slightly worse, 5 - markedly worse). We first calculated the mean of the single-item ratings that belong to one of the three outcome scales (sleep, physical functioning, somatization). This means that the resulting score in direct measures of change is not a single item rating, as it is often used in transition ratings, but is based on the same number of items as the score calculated in indirect or quasi-indirect measures of change. Then we transformed this mean score by subtracting 3, yielding a score that ranged from −2 (worst change possible) to +2 (best change possible). This direct-change score thus has a theoretical range of four scale points and is centred around 0 (no change). The reliability of the status measurements, retrospective pre scores (then-test) and scores for direct measures of change were calculated using Cronbach’s alpha.

The effect size of the change for the direct change measurement (transition rating) was calculated by dividing the mean change-score by its standard deviation. Effect sizes for the indirect and quasi-indirect measures of change were calculated as standardised response means ((M_t1_ − M_t0_)/SD_diff t1-t0_) [24]. In theory, the standard deviation of the transition ratings should represent a standard deviation of a change score. Therefore the standardized response mean that uses the standard deviation of the difference between the scores assessed at of two time points as a denominator should be the most suitable equivalent of the effect size calculated for the transition ratings.

The level of agreement between indirect and quasi-indirect as well as direct measures of change (question 1) was calculated by Pearson product–moment correlation coefficients. The status measures on which the indirect and quasi-indirect measures of change were based were on the same scale. The scale of direct measures of change was different from the scales of indirect and quasi-indirect measures of change. Therefore, we calculated the intra-class correlation coefficient (ICC) between pre test and post test measure used for indirect and quasi-indirect measures of change to analyse the level of absolute agreement of both scales, in addition to the Pearson product–moment correlation coefficient. This was not suitable for levels of agreement or direct measures of change with the other measures of change.

The degree of recall bias (question 2) was estimated using the correlation between the score at t0 and the retrospective pre score assessed at t1 (then-test). A correlation coefficient with a value near the reliability of the two assessments indicates a low recall bias.

The present-state effect (question 3) was analysed according to the approach used by Guyatt et al. [7]. We calculated the correlation between the pre measures and their corresponding transition-rating scores as well as the post measures and their corresponding transition-rating scores. Each transition-rating score was then used as a dependent variable in a linear regression model. We entered the post scores into the regression model first, and then entered the corresponding pre scores subsequently. This procedure allowed us to determine what percentage of variance was explained by the post scores alone and what additional percentage could then be explained using the pre scores. A beta coefficient that is larger for the post score than for the pre score indicates a present-state effect. If a pre score accounts for a substantial amount of variance, it indicates that the status at t1 (the “present state”) does not override the information of the pre status of the patient at t0 which is necessary to make a sound judgement of change.

## Results

### Sample characteristics

Out of 426 participants, 395 (92.7%) completed all questionnaires at both t0 and t1. The characteristics of the study sample are summarised in Table 1. At baseline, the percentage of patients with cardiovascular and musculoskeletal diagnoses was near equal. The majority of the sample were males who tended to be less educated and who generally reported their overall health to be poor. The differences between the group randomised to the then-test and that randomised to direct measurement of change were negligible.

**Table 1:** **Sample characteristics, randomisation and test for group differences at baseline (*n*** **= 395)**

	**Total (*N*** **= 395)**		**Group 1: “direct” (*N*** **= 194)**		**Group 2: “quasi- indirect” (*N*** **= 201)**		**Group difference**^**$**^
	***n***	**%**	***n***	**%**	***n***	**%**	
Female	131	33.2	64	33.0	67	33.3	*p* = .942
Diagnosis							*p* = .865
Cardiovascular	187	47.3	91	46.9	96	47.3	
Musculoskeletal	208	52.7	103	53.1	105	52.7	
Highest level of education completed							*p* = .664
None/elementary school	207	52.8	105	54.7	102	51.0	
Secondary school	101	25.8	49	25.5	52	26.0	
University entrance qualification^#^	77	19.6	36	18.8	41	20.5	
Other	7	1.8	2	1.0	5	2.5	
General health status							*p* = .981
Very good	5	1.3	2	1.0	3	1.5	
Good	38	9.7	18	9.4	20	10.0	
Satisfactory	93	23.7	46	24.0	47	23.4	
Fair	176	44.8	88	45.8	88	43.8	
poor	81	20.6	38	19.8	43	21.4	
	**M**	**SD**	**M**	**SD**	**M**	**SD**	Group difference*
Age	50.5	8.3	50.8	8.2	50.2	8.4	*p* = .449
Physical functioning index (SF-36)	49.1	27.4	47.9	27.1	50.2	27.6	*p* = .403
Somatisation (SCL-90-R)	1.9	0.6	2.0	0.6	1.9	0.5	*p* = .672
Sleep function (IRES)	3.7	1.2	3.8	1.2	3.6	1.2	*p* = .241

Legend: M = mean, SD = standard deviation.

^**$**^*χ*^2^-test, *t-test for independent samples.

^#^ the German “Abitur”.

### Description of change

The means for the pre, retrospective-pre as well as post scores are shown in Table 2 (“status” row). The corresponding reliabilities are presented in Table 3. The absolute levels of change for the different approaches are reported in Table 2 (“change” row). Effect sizes for the physical function index and the somatisation scale were in the clinically relevant range [25].

**Table 2:** Change scores calculated using the different approaches for measuring change (indirect, quasi-indirect, direct)

**Outcome **^**1)**^		**Status**			**Change**		
		**Pre**	**Retro-spective pre**	**Post**	**Indirect post minus pre**	**Quasi-indirect post minus retrospective pre**	**Direct transition rating**
IRES sleep function^2)^	M (SD)	3.7 (1.2)	3.8 (1.3)	4.2(1.1)	0.5 (1.2)	0.4 (1.2)	0.2 (0.7)
	CI 95%	--	--	--	0.4; 0.6	0.2; 0.6	0.1; 0.3
	ES	--	--	--	0.41^3)^	0.36^3)^	0.28^4)^
	*N*	343	192	347	319	161	147
SF-36 physical functioning index^2)^	M (SD)	49.3 (27.3)	48.6 (30.0)	64.3 (26.2)	15.1 (21.5)	16.9 (23.5)	0.5 (0.7)
	CI 95%	--	--	--	12.9; 17.2	13.5; 20.2	0.4; 0.7
	ES	--	--	--	0.70^3)^	0.72^3)^	0.71^4)^
	*N*	383	191	383	383	191	184
SCL-90-R somatisation^5)^	M (SD)	2.0 (0.6)	1.9 (0.6)	1.7 (0.5)	−0.3 (0.5)	−0.2 (0.4)	0.3 (0.5)
	CI 95%	--	--	--	−0.4; −0.3	−0.3; −0.2	0.2; 0.4
	ES	--	--	--	0.66^3)^	0.57^3)^	0.60^4)^
	*N*	386	180	386	386	180	186

Legend: post **−** pre = indirect measurement of change; post **−** retrospective pre = quasi-indirect measurement of change; directly reported change = direct measurement of change; M = mean, SD = standard deviation; CI = 95% confidence interval; ES = effect size.

^1)^ for respondents completing t0 and t1 and providing valid responses^2)^ Higher scores indicate better functioning.

^3)^ standardized response mean (M_t1_ **−** M_t0_)/SD_diff t1−t0_.

^4)^ M/SD.

^5)^ Higher scores indicate higher level of somatisation.

**Table 3:** Correlation between different types of change measurement (indirect, quasi-indirect, direct; product–moment correlation coefficient r or intra-class correlation coeffiecient ICC); regression of transition ratings (standardized linear-regression coefficients); reliability (Cronbach’s alpha)

	**IRES sleep function**	**SF-36 physical-functioning index**	**SCL-90-R somatisation**^**#**^
**Correlation between measures of change**			
Indirect × direct change	*r* = .483***	*r* = .381***	*r* = .375***
Indirect × quasi-indirect change	*r* = .657***	*r* = .713***	*r* = .603***
**Correlation between pre-test, post-test, retrospective pre-test and transition ratings**			
Pre-test × retrospective pre-test	*r* = .682***	*r* = .819***	*r* = .767***
	ICC = .671***	ICC = .815***	ICC = .761***
Pre-test × post-test	*r* = .480***	*r* = .677***	*r* = .612***
Retrospective pre-test × post test	*r* = .521***	*r* = .658***	*r* = .738***
Transition ratings (direct) × pre test	*r* = −.149	*r* = −.129	*r* = −.112
Transition ratings (direct) × post test	*r* = .309***	*r* = .166*	*r* = .229**
**Regression (dependent variable: transition ratings)**			
Post test	*β* = .577***	*β* = .508***	*β* = .486***
Pre test	*β* = −.475***	*β* = −.486***	*β* = −.414***
**Reliability** (Cronbach’s *α*)			
Pre test	*α* = .821	*α =* .919	*α =* .770
Retrospective pre test	*α* = .896	*α =* .938	*α =* .815
Post test	*α* = .817	*α =* .921	*α =* .827
Transition ratings (direct) at post test	*α =* .897	*α =* .959	*α =* .873

^#^ to allow for better comparability increasing numbers refer to improvement of symptoms.

* *p* < .05, ** *p* < .01, ***p < .001.

### Agreement between change measures

Table 3 shows that for all three subscales analysed, the correlation between indirect and quasi-indirect measures of change was found to be substantially higher than the correlation between indirect and direct measures of change.

### Recall bias

The correlation coefficients comparing the scores at t0 to the corresponding retrospective pre score assessed at t1 (then-test) can also be found in Table 3. The correlation coefficient (t0 status and then-test) for the somatisation score was similar to the level of reliabilities of the scales; the correlation coefficients for the sleep scale and the physical functioning scale were also substantial.

### Present-state effect

Direct (i.e. transition) ratings were more correlated to post status than to pre status (Table 3). Results of our regression analysis of direct (i.e. transition) ratings are also presented in Table 3 (standardized regression coefficients). After controlling for post status, we found pre status to be substantially associated to the corresponding transition rating. The amounts of variance accounted for by post status alone, as well as the additional variance accounted for by pre status (i.e. the changes in *R*^2^) were 9.5% and 15.3% (i.e. total *R*^2^ = 24.8%) for the sleep scale, 2.7% and 11.9% for the physical-functioning scale, and 5.2% and 10.2% for the somatisation scale, respectively.

## Discussion

We re-analysed a data set that had originally been collected to investigate the prognostic validity of different approaches to measuring change in the context of rehabilitation treatment^b^. We focused on three self-reported outcome domains (sleep, physical functioning, somatisation) for which the three different approaches to measuring change of interest to us were based on scales with equal numbers of items and equivalent content. To our knowledge, there has only been one other study to analyse the use of a multi-item approach in transition ratings in direct comparison to indirect change measures [15]. Indirect and quasi-indirect change measurements both yielded comparable results measurements, indicating that recall bias does not necessarily affect quasi-indirect change measurements and that the quasi-indirect method has the potential to serve as a substitute for the indirect method (pre-post measurements). Direct change measurement reflects different aspects of change compared to indirect and quasi-indirect change measurements but is not necessarily biased by patients’ present states.

Previous studies have indicated that effect sizes as found using direct change measures are systematically larger than those found using indirect measures of change [13]. This was not the case in our study, however. Therefore, it remains to be shown, in a future head-to-head comparison of general transition items with multi-item transition scales, whether or not the effect reported in previous studies – of direct change measurements overestimating effect sizes – is attributable to the general nature of direct change measures.

Indirect and quasi-indirect measurement of change yielded comparable results in our study. The agreement between pre status and retrospective pre status (“then-test”) was notably high. Thus, for multi-item scales the retrospective pre test might have the potential to measure the same construct as the pre status. Recall bias did not appear to play a major role in this regard. In fact, quasi-indirect assessments are superior to indirect measurements of change in predicting change in physiological indicators in AIDS patients [26]. Quasi-indirect change assessments are not only a feasible approach for estimating the amount of response-shift in quality-of-life studies, but may also come to play an interesting role in clinical studies and quality-assurance programmes. Quasi-indirect measurements are made by asking patients two questions at one time point (after an intervention). In contrast, indirect measurements require contact to be made with the patient two separate times, therefore requiring more resources, and perhaps also causing more patients to drop out. Quasi-indirect and direct measures of change are thus more economical to obtain than indirect measures of change. However, response-shift literature warns us not to be overly optimistic, as no variables have been identified that consistently moderate different degrees of response-shift [5]. Before starting to substitute the quasi-indirect approach of measuring change for the indirect approach in different applications, it is essential to understand these moderating factors. The pre–post interval should be short enough that the patient is able to remember the pre state. In the case of our study, this time interval was about three weeks. Also, a patient should relate his or her responses to a specified point in time which is meaningful to him, e.g. events that are salient to the disease trajectory of a patient. In our study it was the admission to a rehabilitation clinic. To this end, it may prove valuable to use multi-item scales and to avoid single general assessments. A future challenge to research would be to test these moderator variables and also identify additional conditional factors that would allow both indirect and quasi-indirect change measurements to produce equivalent results.

In comparison to other studies [4,27], we found the correlation of direct to indirect measurement of change to be substantive. The correlation coefficients were not as high as those reported by Middle et al. [6] (canonical correlation *R*_c_ = .63) or even Guyatt et al. [7] (correlation coefficients from *r* = .56 to *r* = .82), but higher than the values reported by Kohlmann and Raspe [4] (correlation coefficients from *r* = .10 to *r* = .37). The strength of this relationship might be interpreted as an indication that both direct and indirect measurement approaches capture the same change process, albeit different aspects of it.

Various studies have reported direct ratings to be more strongly related to a patient’s status at the time of measurement than to change as assessed using indirect measures, a phenomenon which is referred to as present-state bias [8,15]. These findings, if true, imply that direct measurements of change are highly influenced by a patient’s state at the time of measurement and that these direct measurements are only minimally influenced by those aspects of change that are reflected in indirect measurements. Applying the analytical approach used by Guyatt et al. [7], we were able to show, as expected, that performing a regression of transition ratings on post status and pre status yielded beta parameters of the post status and pre status of inverse signs and similar magnitudes – although the betas for the pre-status variables were slightly lower than the post-status variables, as has been reported in other studies [7]. The amount of variance accounted for by the post scores was below 10% in all three outcome domains, while adding the pre score increased the amount of variance accounted for from 10% to 15%. While these results indicate that transition ratings are not necessarily dominated by the present state of the respondent, it has to be acknowledged that pre- and post-status scores were unable to account for a substantial amount of variance in transition ratings. It is therefore necessary to identify additional explanatory variables to further our understanding of transition ratings.

A major limitation of our study design is that it did not allow for a head-to-head comparison between quasi-indirect and direct measurements of change, nor did it allow for a head-to-head comparison between multi-item approaches and general approaches for measuring change directly or (quasi-) indirectly. Also, the study design led to there being fewer data points for the direct and quasi-indirect approaches than for the indirect approach (*cf*. Table 2).

Two biases might have unduly elevated the (unusually strong) correlation between indirect and quasi-indirect measures of change. First, our then-test might be prone to a memory bias if patients were able to remember how they rated the different items at t0 and if they had tried to present themselves accordingly, i.e. they might have attempted to make their current (t1) ratings of their to condition correspond to how they rated them before. Omitting the status assessment at t0 would eliminate any pre testing effects, such as patients’ deliberately making their answers on the retrospective pre test correspond to those they had given on the actual pre-test, or perhaps patients’ being sensitized to particular changes. However, omitting the pre assessment would still not allow for a head-to-head comparison between indirect and quasi-indirect measures of change. Nevertheless, we do not believe this kind of recall to be a major threat to validity because in our experience most patients have substantial difficulties remembering even important activities and interactions from the admission phase, which is likely due in part to how overwhelming rehabilitation appears to be for patients at the start of rehabilitation. This does not preclude that the patients are able to remember their health status at the time around their submission. It is reasonable to assume that this information is more general in nature compared to marks on a questionnaire, and it is inextricably linked to a patient’s reason for applying to medical rehabilitation or to his or her perception of the course of illness. A second bias is due to the fact that both indirect and quasi-indirect measures of change rely on the same post-status score, which systematically increases the association between these two variables. However, it did not seem reasonable to carry out two independent post-status assessments. Therefore, this second bias cannot be eliminated. Furthermore, estimating its magnitude is not possible given the current study design.

As Table 2 shows, the sleep-function scale yielded considerably more missing values than the other two scales did. There is no obvious reason for why this would be the case. One possible reason could be the ‘checklist misconception effect’ [28]: subjects might have misunderstood the items of the sleep-function scale as a checklist, in which ‘true’ items were to be checked (thereby confirming that they experienced these symptoms) whereas items that did not apply were to be left blank. Controlling for the checklist misconception effect in our analysis did not however substantially affect the results (data not shown).

We chose not to classify the results of the transition-rating scales into clinically meaningful categories. We avoided making such decisions for two reasons: First, we would need to know how large to make the ‘region of indifference’ – i.e. how small empirical differences would have to be for them to be regarded as too minor to merit classifying the patients as having changed – or which thresholds for change to use in general. Second, further research is needed on the question of whether these thresholds should be symmetrical around the point of indifference, i.e. whether they should be equidistant from zero in both positive and negative directions.

## Conclusions

Quasi-indirect measurement of change has the potential to serve as a substitute for indirect measurement of change. It appears to be a suitable assessment method in situations where no baseline assessments are possible, especially non-elective care situations. However, further exploration is needed into potential moderating factors and their implications. Also, the correlation between quasi-indirect and indirect change scores might be spuriously strong due to the fact that the post-test measurement is used to compute both indirect and quasi-indirect change scores.

Transition ratings measure different aspects of change than indirect measurement of change do. We still need a comprehensive model of what transition ratings actually measure. Research making use of qualitative methodology or cognitive interviewing techniques may prove to be valuable in identifying important factors of such a model, as has been suggested by Nieuwkerk et al. [26].

## Endnotes

^a^It should be noted that we are not using the term ‘agreement’ here in the strict sense of perfect equivalence, since direct and indirect approaches to measure change are both based on different scales.

^b^This study was not able to find any advantage to one of these approaches to measuring change over the other; this result has not been published.

## Competing interests

The authors declare that they have no competing interests.

## Authors’ contributions

TM developed the idea for the re-analysis presented here, conducted parts of that analysis, and drafted the paper in close cooperation with SR. SR developed the idea for the re-analysis presented here, conducted parts of that analysis, and drafted the paper in close cooperation with TM. HR supervised the analysis in the original study, developed the general idea of comparing the different approaches to measuring change, and critically revised a draft of the article. All authors read and approved the final manuscript.

## Pre-publication history

The pre-publication history for this paper can be accessed here:

http://www.biomedcentral.com/1471-2288/13/52/prepub
